# 化疗间插联合EGFR-TKIs对比单独化疗一线治疗晚期非小细胞肺癌的*meta*分析

**DOI:** 10.3779/j.issn.1009-3419.2016.12.06

**Published:** 2016-12-20

**Authors:** 超煜 洪, 同华 梅, 进 王

**Affiliations:** 400016 重庆，重庆医科大学附属第一医院呼吸内科 Department of Respiratory Medicine, First Affiliated Hospital of Chongqing Medical University, Chongqing 400016, China

**Keywords:** 肺肿瘤, 化疗, 表皮生长因子受体酪氨酸激酶抑制剂, 间插联合疗法, 一线治疗, *Meta*分析, Lung neoplasms, Chemotherapy, Epidermal growth factor receptor-tyrosine kinase inhibitors, Intercalated combination, First-line treatment, *Meta*-analysis

## Abstract

**背景与目的:**

化疗与表皮生长因子受体酪氨酸激酶抑制剂(epidermal growth factor receptor-tyrosine kinase inhibitors, EGFR-TKIs)联合疗法一直是许多研究的焦点，其中间插联合疗法受到了更多研究者的关注。本研究旨在系统评价化疗与EGFR-TKIs间插联合疗法对比单独化疗一线治疗晚期非小细胞肺癌(non-small cell lung cancer, NSCLC)的有效性及安全性。

**方法:**

检索The Cochrane Library、PubMed、EMBASE、中国生物医学文献数据库(CBM)、知网和万方等数据库关于化疗间插联合EGFR-TKIs疗法和单独化疗一线治疗晚期NSCLC的随机对照试验(randomized controlled trial, RCT)，分析如下结局指标：无进展生存期(progression-free survival, PFS)、总体生存期(overall survival, OS)、客观缓解率(objective response rate, ORR)、疾病控制率(disease control rate, DCR)以及不良反应发生率。由两名研究者根据Cochrane系统评价手册筛选文献、进行质量评价以及提取并交叉核对数据。应用Stata12.0软件进行*meta*分析。

**结果:**

本研究共纳入6个RCT，共计933例晚期NSCLC患者。*Meta*分析结果表明，在晚期NSCLC患者一线治疗中，与单独化疗相比，间插联合疗法虽然延长了患者的PFS(HR=0.72, 95%CI: 0.53-0.98, *P*=0.037)，但并不能提高其OS(HR=0.85, 95%CI: 0.72-1.01, *P*=0.060)、ORR(OR=1.59, 95%CI: 0.86-2.95, *P*=0.142)和DCR(OR=1.09, 95%CI: 0.95-1.25, *P*=0.226)。进一步的亚组分析发现，间插联合疗法提高了女性、腺癌、从不吸烟和EGFR突变等患者的PFS，差异具有统计学意义。在安全性方面，间插联合疗法的主要不良反应为皮疹(OR=7.81, 95%CI: 3.74-16.34, *P* < 0.001)和腹泻(OR=2.73, 95%CI: 1.92-3.89, *P* < 0.001)。

**结论:**

一线接受化疗间插联合EGFR-TKIs治疗的NSCLC患者的PFS明显高于接受单独化疗者，其主要不良事件是皮疹和腹泻。因此，间插联合治疗具有一定优势，但仍需要更多大样本、高质量的RCT进一步验证。

## Abstract (en)

**Background and objective:**

The combination therapy of chemotherapy and epidermal growth factor receptor-tyrosine kinase inhibitors (EGFR-TKIs) has attracted the attention of more and more investigators. The aim of this *meta*-analysis is to evaluate the clinical efficacy and safety of intercalated combination of chemotherapy and EGFR-TKIs versus chemotherapy alone in the first-line therapy of advanced non-small cell lung cancer (NSCLC).

**Methods:**

We retrieved the Cochrane Library, PubMed, EMBASE, CBM, CNKI and Wanfang databases for randomized controlled trials which involved the intercalated combination of chemotherapy and EGFR-TKIs, and chemotherapy alone in the first-line treatment of advanced NSCLC. The progression-free survival (PFS), overall survival (OS), objective response rate (ORR), disease control rate (DCR), and adverse events were analyzed. The quality evaluation and cross-checked data were independently performed by two investigators according to the Cochrane Systematic Reviews Handbook. The Stata 12.0 software was used to conduct the *meta*-analysis.

**Results:**

This study included 933 NSCLC patients from 6 RCTs. The *meta*-analysis demonstrated that the intercalated combination of chemotherapy and EGFR-TKIs significantly prolonged the PFS (HR=0.72, 95%CI: 0.53-0.98, *P*=0.037) of advanced NSCLC patients compared with mono-chemotherapy. However, there was no statistical difference in OS (HR=0.85, 95%CI: 0.72-1.01, *P*=0.060), ORR (OR=1.59, 95%CI: 0.86-2.95, *P*=0.142) and DCR (OR=1.09, 95%CI: 0.95-1.25, *P*=0.226) between the two groups. Further, the subgroup analysis showed that the intercalated combination markedly improved the PFS in female, adenocarcinoma, never smoking, EGFR mutant patients. In the aspect of safety, the main side effects of the intercalated combination therapy were rash (OR=7.81, 95%CI: 3.74-16.34, *P* < 0.001) and diarrhea (OR=2.73, 95%CI: 1.92-3.89, *P* < 0.001).

**Conclusions:**

The intercalated combination of chemotherapy and EGFR-TKIs significantly prolonged the PFS in the first-line therapy of advanced NSCLC patients compared with mono-chemotherapy, and the main adverse events were tolerable rash and diarrhea. Together, the intercalated combination shows promising results, and more large-scale and high-quality RCTs are still needed.

肺癌是世界范围内癌症相关死亡最常见的原因^[1]^，其中大约85%-90%的肺癌患者是非小细胞肺癌(non-small cell lung cancer, NSCLC)。然而，大部分患者被诊断时已经是晚期，无法进行手术切除^[2]^。因此，晚期NSCLC患者主要采取药物治疗。在靶向药物尚未问世前，含铂双药化疗是晚期NSCLC标准的一线治疗方案。近年来，随着肺癌发病机制的进一步研究，表皮生长因子受体酪氨酸激酶抑制剂(epidermal growth factor receptor-tyrosine kinase inhibitors, EGFR-TKIs)开始一线用于*EGFR*基因突变的NSCLC患者^[3-5]^。为了进一步提高NSCLC患者的生存获益，含铂双药化疗联合EGFR-TKIs已经成为许多研究的新焦点。在过去的十年，若干个研究评价了EGFR-TKIs同步联合标准化疗在晚期NSCLC患者中的疗效。结果表明，EGFR-TKIs同步联合标准化疗和单独化疗对比并不能提高患者的生存获益^[6-9]^，主要原因是化疗药物和EGFR-TKIs之间存在拮抗性，从而降低了疗效^[10]^。

为此，人们对化疗和EGFR-TKIs的联合方式进行了调整，采取间插联合的方式。FASTACT-1研究^[11]^报道了在化疗周期内应用EGFR-TKIs对比单独化疗能延长无进展生存期(progression-free survival, PFS)，但总体生存期(overall survival, OS)和客观缓解率(objective response rate, ORR)并没有提高。然而，FASTACT-2研究^[12]^表明，化疗间插联合EGFR-TKIs对比单独化疗不但能提高OS和PFS，而且能够提高肿瘤缓解率。同样，国内过雪丹等^[13]^的研究结果与FASTACT-2的报道结果相似。但是随后的一系列临床研究^[14-16]^结果与FASTACT-2完全相反。基于上述临床研究结果，我们进行了该*meta*分析评估一线接受化疗间插联合EGFR-TKIs对比单独化疗在晚期NSCLC患者中的有效性及安全性，探索是否一线化疗间插联合EGFR-TKIs优于单独化疗，为晚期NSCLC的治疗提供更优选择。

## 1 资料与方法

### 1.1 检索策略

由两名研究者采用主题词与自由词联合检索的方法分别检索The Cochrane Library、PubMed和EMBASE等英文数据库以及CBM、知网和万方等中文数据库，检索时间自1965年1月-2016年2月。检索化疗间插联合EGFR-TKIs对比单独化疗一线治疗晚期NSCLC的RCT。英文检索词为“NSCLC”、“non-small cell lung cancer”、“EGFR-TKIs”、“intercalated”、“Chemotherapy”、“first-line treatment”、“Randomized Controlled Trial”等；中文检索词为“非小细胞肺癌”、“表皮生长因子受体酪氨酸激酶抑制剂”、“间插”、“化疗”、“一线治疗”、“随机对照试验”等。同时扩大检索纳入文献的参考文献。

### 1.2 纳入和排除标准

① 研究对象：年龄≥18岁，经病理学证实为初治的晚期NSCLC患者，临床分期为Ⅲb期/Ⅳ期，体力状况评分(performance status, PS)≤2分，无绝对化疗禁忌症。②干预措施：实验组采用化疗间插联合EGFR-TKIs，对照组采用单独化疗。③研究类型:公开发表的前瞻性随机对照试验。④纳入研究提供以下结局指标：PFS、OS、ORR、疾病控制率(disease control rate, DCR)和不良反应发生率。⑤当存在重复研究时，取数据最完整的研究。⑥排除合并第二个恶性肿瘤者以及数据无法进行分析的研究。

### 1.3 文献筛选和数据提取

由两名研究者根据文献纳入和排除标准，首先对文题和摘要进行初筛，其次通过阅读全文进行排除，意见不统一时通过讨论或第三方解决。主要提取以下数据: ①一般信息：题目、第一作者、发表年份。②研究对象的临床特征，如性别、年龄、分期、病理类型、吸烟状况和*EGFR*基因突变状况等。③干预措施：化疗间插联合EGFR-TKIs方案、单独化疗方案。④各种结局指标：PFS、OS、ORR、DCR和不良反应发生率等。

### 1.4 质量评价

由两名研究者按Cochrane系统评价手册5.1.0质量评价标准^[17]^从随机分配方法、分配隐藏、盲法、不完整数据报告、选择性发表及其他偏倚来源等6个方面对纳入文献交叉进行质量评价。对每一项研究结果，分别按照上述6条内容做出“是”(低风险)、“否”(高风险)和“不清楚”(风险未知)的判断。完全满足上述6条质量标准，其发生各种偏倚的可能性最小，质量为A级；≥上述1条描述不清楚者，有发生相应偏倚的中度可能性，质量为B级；≥上述1条未描述者有发生相应偏倚的高度可能性，质量为C级。

### 1.5 统计学方法

统计学分析采用Stata 12.0软件，分别计算PFS和OS的合并HR值，ORR、DCR和不良事件的合并OR值，同时计算HR值和OR值的95%置信区间。采用Q检验检测纳入研究间的统计学异质性，同时采用*I*^2^统计量评价异质性大小。如果*P* > 0.05、*I*^2^ < 50%时，采用固定效应模型，反之采用随机效应模型。如果异质性明显，则进行亚组分析探索异质性的来源，必要时采用敏感性分析检验结果的稳定性。若异质性过大无法进行*meta*分析，则只做一般性描述。*P* < 0.05为差异有统计学意义。

## 2 结果

### 2.1 检索结果

共检索到630篇文献。首先排除重复文献210篇，再经阅读文题和摘要排除动物研究、*meta*分析、综述、单臂研究以及个案报道共378篇，最后阅读全文排除数据不完整的RCT，共6篇文章^[11-16]^最终纳入该*meta*分析。文献的筛选流程如图 1。

**1:**
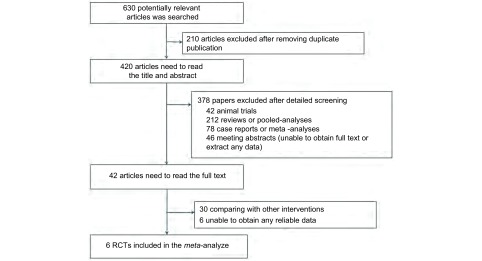
文献检索及筛选流程图 Flow of identification and inclusion of trials

### 2.2 纳入研究的基本信息和质量评价结果

6个研究共纳入933例患者，其中联合治疗组有465例，单独化疗组有468例。3个研究^[12, 14, 16]^提供了*EGFR*突变状况，3个研究^[11, 12, 14]^提供了PFS亚组分析数据，3个研究^[11, 12, 15]^的试验组使用了厄洛替尼，具体纳入文献的特征见表 1。同时采用Cochrane系统评价手册5.1.0质量评价标准^[17]^从随机分配方法、分配隐藏、盲法、不完整数据报告、选择性发表及其他偏倚来源等6个方面对纳入文献进行质量评价(表 2)。

**1:** 纳入文献的基本特征 The basic characteristics of included trials

Trials	Intercalated combination group					Mono-chemotherapy group			
	Case	Interventions	Median OS (mo)/ Median PFS (mo)	ORR (%)/ DCR (%)		Case	Interventions	Median OS (mo)/ Median PFS (mo)	ORR (%)/ DCR (%)
Tony S.K. Mok, 2009	76	Gem 1, 250 mg/m^2^ (d1, d8)+Cis 75 mg/m^2^ (d1)+Erl 150 mg/d (d15-28)	17.20/6.83	35.50/80.30		78	Gem 1, 250 mg/m^2^ (d1, d8)+Cis 75 mg/m^2^ (d1)+Pla (d15-d28)	17.60/5.44	24.40/76.90
Guo xue dan, 2012	36	Gem 1, 000 mg/m^2^ (d1, d8)+Cis 25 mg/m^2^ (d1-d4)+Gef 250 m/d (d10-d24)	12.10/7.30	36.10/83.30		35	Gem 1, 000 mg/m^2^ (d1, d8)+Cis 25 mg/m^2^ (d1-d4)	10.80/5.80	14.30/42.90
Yi-Long Wu, 2013	226	Gem 1, 250 mg/m^2^ (d1, d8)+Cis 75 mg/m^2^ (d1)+Erl 150 mg/d (d15-d28)	18.30/7.60	42.90/80.50		225	Gem 1, 250 mg/m^2^ (d1, d8)+Cis 75 mg/m^2^ (d1)+Pla 150 mg/d (d15-d28)	15.20/6.00	18.20/79.60
Hui yu, 2014	58	Pem 500 mg/m^2^ (d1)+Cis 75 mg/m^2^ (d1)+Gef 250 mg/d (d3-d16)	25.40/7.90	50.00/84.50		59	Pem 500 mg/m^2^ (d1)+Cis 75 mg/m^2^ (d1)	20.80/7.00	47.40/83.10
Michael MICHAEL, 2015	26	Gem 1, 250 mg/m^2^ (d1, d8)+Erl 150 mg/d (d15-d28)	NA/2.40	3.80/38.50		28	Gem 1, 000 mg/m^2^ (d1, d8, d15)	NA/1.86	7.10/50.00
Yoon Ji Choi, 2015	43	Pac 175 mg/m^2^ (d1)+Car AUC 5 (d1)+Gef 250 mg (d2-d15)	9.30/4.10	41.90/74.40		43	Pac 175 mg/m^2^ (d1)+Car AUC 5 (d1)	10.50/4.10	39.50/65.10
Gem: gemcitabine; Cis: cisplatin; Erl: erlotinib; Pla: placebo; Gef: gefitinib; Pem: pemetrexed; Pac: paclitaxel; Car: carboplatin; NA: not available; AUC: area under curve.									

**2:** 纳入文献的质量评价 Quality evaluation of included trials

Trials	Random sequence generation	Allocation concealment	Blindness	Incomplete outcome data	Selective reporting	Other sources of bias	Literature quality
Tony S.K. Mok, 2009	Yes	Yes	Unclear	Yes	Yes	Yes	B
Guo xue dan, 2012	Yes	No	No	Yes	Yes	Yes	C
Yi-Long Wu, 2013	Yes	Unclear	Yes	Yes	Yes	Yes	B
Hui yu, 2014	Yes	No	Unclear	Yes	Yes	Yes	C
Michael MICHAEL, 2015	Yes	Yes	Unclear	Yes	Yes	Yes	B
Yoon Ji Choi, 2015	Yes	Yes	Unclear	Yes	Yes	Yes	B

### 2.3 *Meta*分析结果

#### 2.3.1 PFS

共5篇文献^[11, 12, 14-16]^报道了PFS，各研究间异质性明显(*I*^2^=68.8%, df=4, *P*=0.012)，故采用随机效应模型。结果显示，间插联合能提高PFS(HR=0.72, 95%CI: 0.53-0.98)，具有统计学差异(*Z*=2.08, *P*=0.037)。由于异质性明显，故分别根据EGFR-TKIs、化疗方案和文献质量分级进行亚组分析，结果显示，①在EGFR-TKIs的亚组分析中，吉非替尼组的异质性消失(*I*^2^=0, df=1, *P*=0.835)，厄洛替尼组的异质性下降不明显(*I*^2^=67.1%, df=2, *P*=0.048)(图 2)；②在化疗方案的亚组分析中，吉西他滨组的异质性无明显下降(*I*^2^=67.1%, df=2, *P*=0.048)(图 3)；③在文献质量分级的亚组分析中，文献质量为B级的亚组异质性无明显变化(*I*^2^=71.4%, df=3, *P*=0.015)(图 4)。同时对两组在性别、年龄、临床分期、病理类型、吸烟状况以及*EGFR*突变状况等方面进行亚组分析，除了男性和*EGFR*突变未知型两个亚组的异质性明显，采取随机效应模型进行*meta*分析外，其余各个亚组同质性好，故采用固定效应模型。结果显示，间插联合疗法提高了年龄 < 65岁、女性、Ⅲb期、Ⅳ期、腺癌、从不吸烟和*EGFR*突变等患者的PFS，差异具有统计学意义(表 3)。

**3:**
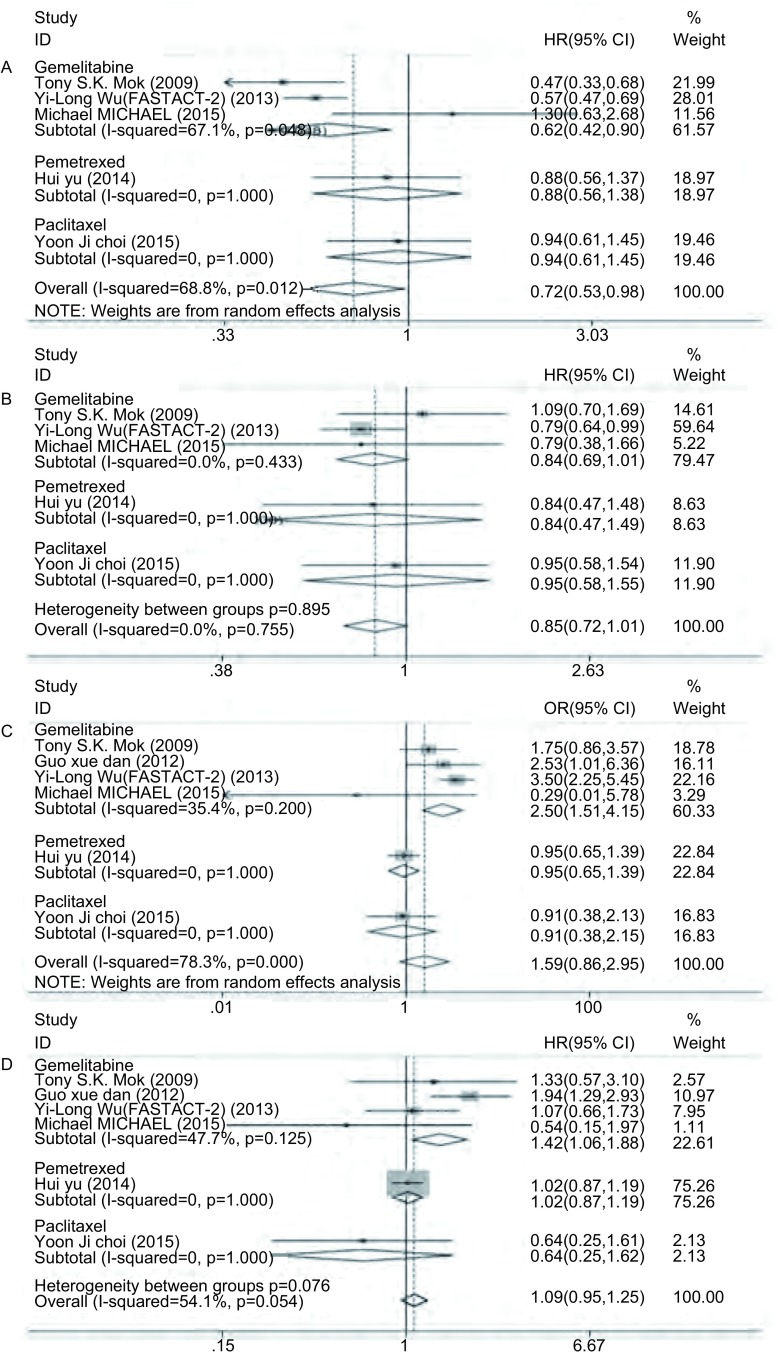
按化疗方案行亚组分析森林图。A：PFS；B：OS；C：ORR；D：DCR。 Forest plot of subgroup analysis according to different chemotherapy. A: PFS; B: OS; C: ORR; D: DCR.

**4:**
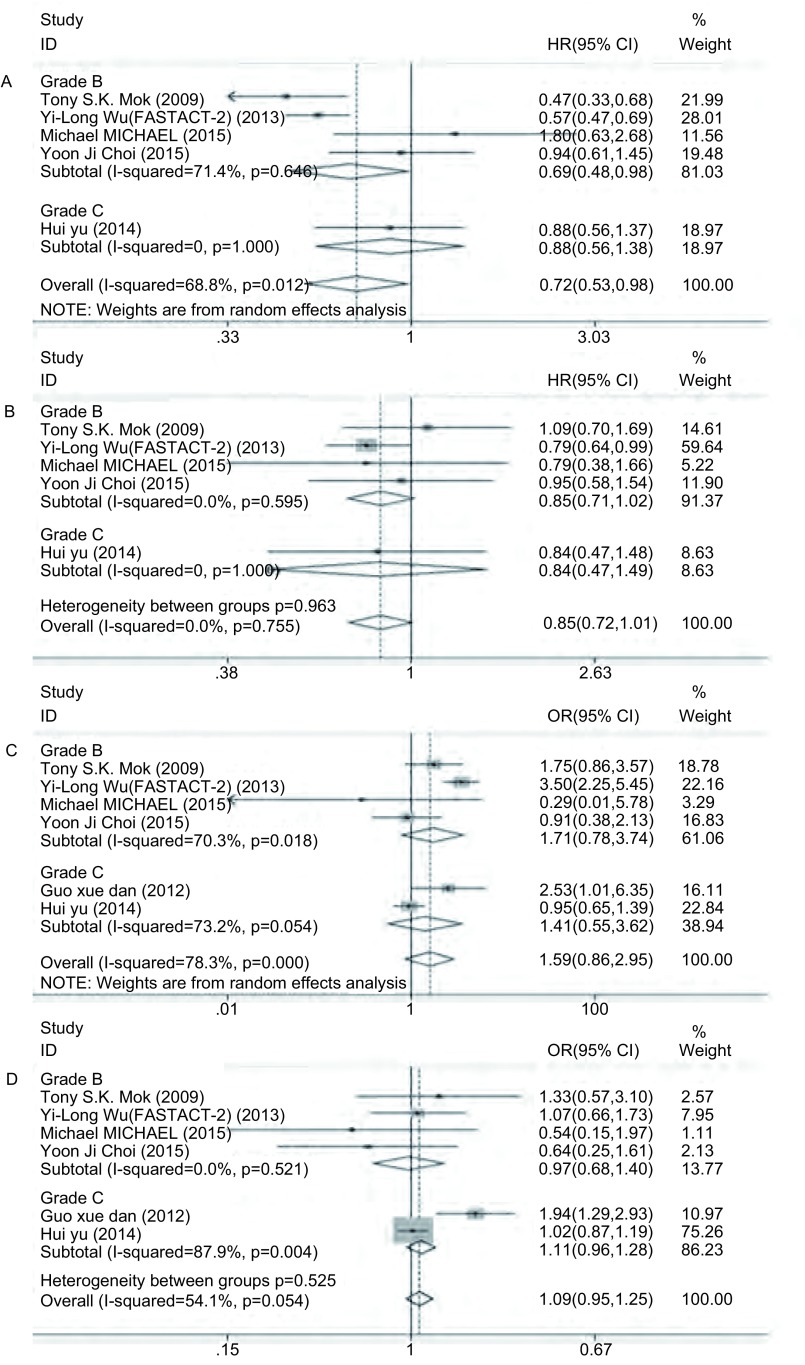
按文献质量分级行亚组分析森林图。A：PFS；B：OS；C：ORR；D：DCR。 Forest plot of subgroup analysis according to study quality. A: PFS; B: OS; C: ORR; D: DCR.

**3:** 间插联合组和单独化疗组的PFS亚组分析结果 Subgroup analysis of PFS between treatment group and control group

Subgroup	Study	HR (95%CI)	Heterogeneity test	Overall effect test
Age < 65 yr	Tony S.K. Mok (2009)	0.49 (0.33-0.74)	*I*^2^=0, *P* > 0.999	HR=0.49 (0.40-0.60) *P* < 0.001
	Yi-Long Wu (2013)	0.49 (0.39-0.61)		
Age≥65 yr	Tony S.K. Mok (2009)	0.72 (0.37-1.39)	*I*^2^=0, *P*=0.656	HR=0.82 (0.57-1.16) *P*=0.263
	Yi-Long Wu (2013)	0.86 (0.57-1.31)		
Male	Tony S.K. Mok (2009)	0.52 (0.35-0.79)	*I*^2^=68.3%, *P*=0.076	HR=0.67 (0.44-1.01) *P*=0.056
	Yi-Long Wu (2013)	0.80 (0.63-1.03)		
Female	Tony S.K. Mok (2009)	0.55 (0.30-1.03)	*I*^2^=43.7%, *P*=0.183	HR=0.38 (0.28-0.52) *P* < 0.001
	Yi-Long Wu(2013)	0.34 (0.24-0.48)		
Ⅲb stage	Tony S.K. Mok (2009)	0.29 (0.11-0.76)	*I*^2^=0, *P*=0.335	HR=0.43 (0.24-0.75) *P*=0.003
	Yi-Long Wu (2013)	0.52 (0.26-1.03)		
Ⅳ stage	Tony S.K. Mok (2009)	0.57 (0.39-0.83)	*I*^2^=0, *P* > 0.999	HR=0.57 (0.48-0.68) *P* < 0.001
	Yi-Long Wu(2013)	0.57 (0.47-0.71)		
Adenocarcinoma	Tony S.K. Mok (2009)	0.48 (0.31-0.74)	*I*^2^=0, *P*=0.870	HR=0.50 (0.41-0.61) *P* < 0.001
	Yi-Long Wu (2013)	0.50 (0.40-0.63)		
Nonadenocarcinoma	Tony S.K. Mok (2009)	0.66 (0.37-1.18)	*I*^2^=0, *P*=0.402	HR=0.81 (0.59-1.12) *P*=0.204
	Yi-Long Wu (2013)	0.89 (0.60-1.31)		
Previous smoking	Tony S.K. Mok (2009)	0.55 (0.26-1.15)	*I*^2^=11.4%, *P*=0.288	HR=0.78 (0.55-1.12) *P*=0.178
	Yi-Long Wu (2013)	0.87 (0.58-1.30)		
Never smoking	Tony S.K. Mok (2009)	0.37 (0.20-0.71)	*I*^2^=0, *P*=0.515	HR=0.42 (0.33, 0.54) *P* < 0.001
	Yi-Long Wu (2013)	0.40 (0.30-0.54)		
	Hui yu (2014)	0.57 (0.32-1.02)		
*EGFR*-mutant	Yi-Long Wu (2013)	0.25 (0.16-0.39)	*I*^2^=0, *P*=0.759	HR=0.24 (0.16, 0.37) *P* < 0.001
	Hui yu (2014)	0.20 (0.05-0.75)		
*EGFR*-wild	Yi-Long Wu (2013)	0.97 (0.69-1.36)	*I*^2^=0, *P*=0.797	HR=0.95 (0.71, 1.27) *P*=0.718
	Hui yu (2014)	0.89 (0.51-1.57)		
*EGFR*-unknown	Yi-Long Wu (2013)	0.61 (0.46-0.82)	*I*^2^=85.3%, *P*=0.009	HR=1.07 (0.31, 3.76) *P*=0.911
	Hui yu (2014)	2.21 (0.88-5.57)		

**2:**
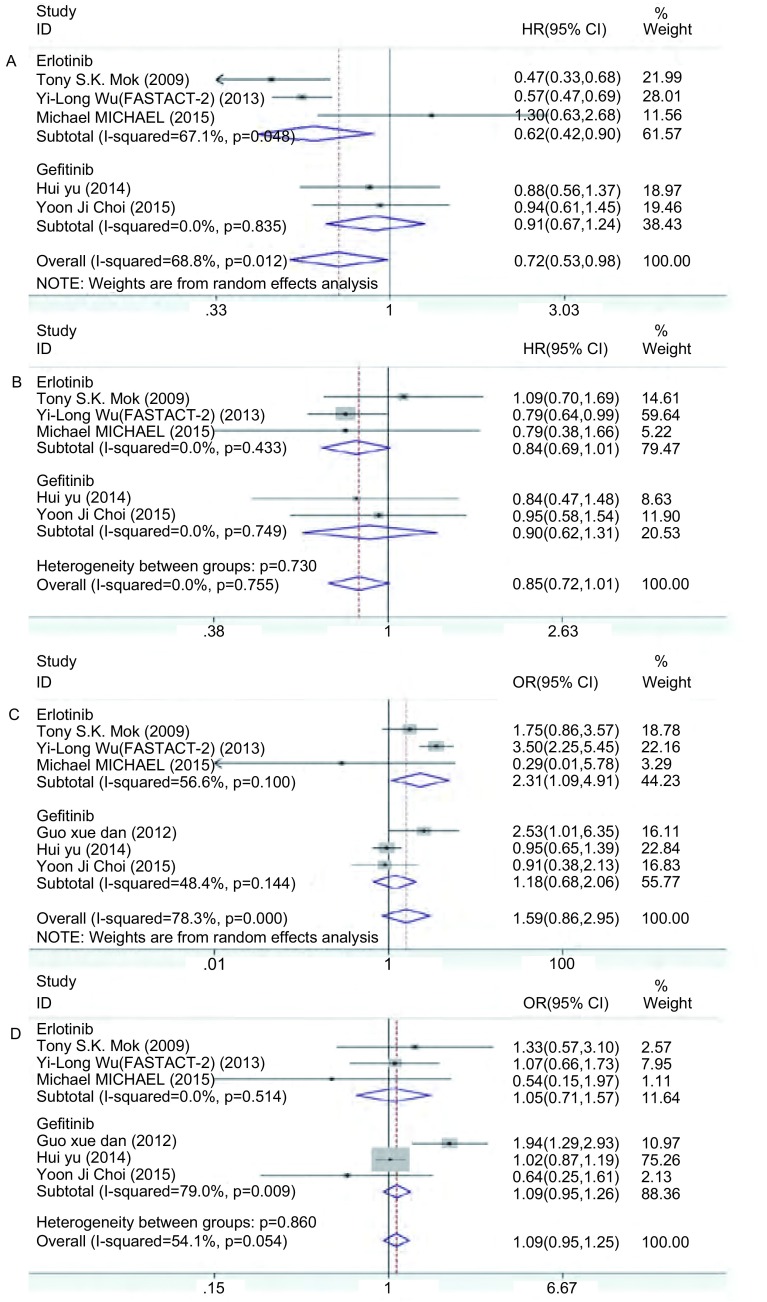
按EGFR-TKI行亚组分析森林图。A：PFS；B：OS；C：ORR；D：DCR。 Forest plot of subgroup analysis according to different EGFR-TKI. A: PFS; B: OS; C: ORR; D: DCR. PFS: progression free survival; OS: overall survival; ORR: objective response rate; DCR: disease control rate.

#### 2.3.2 OS

共5篇文献^[11, 12, 14-16]^报道了OS。各研究间同质性好(*I*^2^=0, df=4, *P*=0.755)，故采用固定效应模型，同时进行亚组分析。结果显示，化疗间插联合EGFR-TKIs较单独化疗在提高OS方面无优势(HR=0.85, 95%CI: 0.72-1.01)，不具有统计学差异(*Z*=1.88, *P*=0.060)，而两者在EGFR-TKIs(*P*=0.730)、化疗方案(*P*=0.895)和文献质量分级(*P*=0.963)的亚组分析中均具有较好同质性。

#### 2.3.3 ORR

共6篇文献^[11-16]^报道了ORR。各研究间异质性明显(*I*^2^=78.3%, df=5, *P* < 0.001)，故采用随机效应模型。结果显示，化疗间插联合EGFR-TKIs较单独化疗并不能提高ORR(OR=1.59, 95%CI: 0.86-2.95)，不具有统计学差异(*Z*=1.47, *P*=0.142)。由于两组间存在异质性，故进行亚组分析，结果显示，①在EGFR-TKIs的亚组分析中，吉非替尼组(*I*^2^=48.4%, df=2, *P*=0.144)和厄洛替尼组(*I*^2^=56.6%, df=2, *P*=0.100)的异质性均明显下降，见图 2；②在化疗方案的亚组分析中，吉西他滨组的异质性下降(*I*^2^=35.4%, df=3, *P*=0.200)，培美曲塞组和紫杉醇组无法比较(图 3)；③在文献质量分级的亚组分析中，评级为“B”(*I*^2^=70.3%, df=3, *P*=0.018)和“C”(*I*^2^=73.2%, df=1, *P*=0.054)的亚组异质性无明显变化(图 4)。

#### 2.3.4 DCR

共6篇文献^[11-16]^报道了DCR。各研究间异质性不明显(*I*^2^=54.1%, df=5, *P*=0.054)，故采用固定效应模型，同时进行亚组分析。结果显示，化疗间插联合EGFR-TKIs组对比单独化疗组在DCR方面无优势(OR=1.09, 95%CI: 0.95-1.25)，不具有统计学差异(*Z*=1.21, *P*=0.226)。亚组分析显示，①在EGFR-TKIs的亚组分析中，吉非替尼组异质性增大(*I*^2^=79.0%, df=2, *P*=0.009)，而厄洛替尼组的异质性消失(*I*^2^=0, df=2, *P*=0.514)(图 2)；②在化疗方案的亚组分析中，吉西他滨组的异质性下降(*I*^2^=47.7%, df=3, *P*=0.125)，而各种化疗方案之间的异质性不明显(*P*=0.076)(图 3)；③在文献质量分级的亚组分析中，评级为“B”的亚组同质性好(*I*^2^=0, df=3, *P*=0.521)，但“B”级和“C”级亚组之间无明显异质性(*P*=0.525)(图 4)。

### 2.4 不良事件

6个研究中发生率较高的不良事件包括皮疹、腹泻、恶心、疲乏、厌食、贫血、血小板减少和中性粒细胞减少等，其中在皮疹方面，各研究间的异质性明显，采用随机效应模型，其余不良事件中各研究间同质性好，故采用固定效应模型。结果表明，间插联合组的皮疹(OR=7.81, 95%CI: 3.74-16.34, *P* < 0.001)和腹泻(OR=2.73, 95%CI: 1.92-3.89, *P* < 0.001)发生率较单独化疗组高，具有统计学差异，其余不良事件在两组间的发生率相似。两组不良事件比较见表 4。

**4:** 间插联合组和单独化疗组不良事件*meta*分析结果 *Meta*-analysis of adverse event between treatment group and control group

Adverse event	Heterogeneitytest	Overall effect test
Rash	*I*^2^=71.2%, df=5, *P*=0.004	OR=7.81, 95%CI: 3.74-16.34, *P* < 0.001
Diarrhea	*I*^2^=31.4%, df=5, *P*=0.200	OR=2.73, 95%CI: 1.92-3.89, *P* < 0.001
Nausea	*I*^2^=16.1%, df=4, *P*=0.312	OR=1.02, 95%CI: 0.78-1.35, *P*=0.873
Fatigue	*I*^2^=0, df=4, *P*=0.550	OR=1.12, 95%CI: 0.82-1.53, *P*=0.473
Anorexia	*I*^2^=3.5%, df=4, *P*=0.386	OR=0.93, 95%CI: 0.70-1.23, *P*=0.591
Anemia	*I*^2^=7.8%, df=4, *P*=0.362	OR=1.01, 95%CI: 0.76-1.34, *P*=0.958
Thrombopenia	*I*^2^=0, df=4, *P*=0.800	OR=0.90, 95%CI: 0.63-1.29, *P*=0.575
Neutropenia	*I*^2^=0, df=4, *P*=0.805	OR=1.01, 95%CI: 0.75-1.35, *P*=0.946

## 3 结论

肺癌约85%-90%是NSCLC，在过去二十年，晚期NSCLC标准的一线治疗方案是含铂的双药化疗，但是一线标准化疗的临床疗效已经到达平台期^[18]^，因此需要寻找新的治疗策略。近年来，随着肺癌发病机制的进一步研究，一线推荐在*EGFR*基因突变的NSCLC患者中使用EGFR-TKIs，尤其是厄洛替尼和吉非替尼，这一治疗策略的可行性已经在多个大型随机对照试验中得到验证。然而，这些靶向药物的临床优势只限制在*EGFR*基因突变的患者，且不可避免发生耐药。因此，许多研究都致力于通过联合以铂类为基础的标准化疗和EGFR-TKIs来扩大适应证和提高疗效，包括同步联合、维持治疗和间插联合等三种策略。

然而，四个大型三期临床研究，包括TRIBUTE、TALENT、INTACT-Ⅰ和INTACT-Ⅱ^[6-9]^已经证明，第一种治疗策略，即化疗同步联合EGFR-TKIs较单独化疗并不能在晚期NSCLC患者中带来更多临床获益。可能原因有以下几种：①这种药物同步联合方式存在潜在的拮抗作用，因为临床前期研究已经表明EGFR-TKIs能诱导癌细胞停滞于G_1_细胞周期，而处于G_1_细胞周期的癌细胞对化疗的敏感性差；②同步联合方式有可能导致化疗作用掩盖EGFR-TKIs的作用；③化疗有可能对EGFR的表达和功能产生影响，导致EGFR-TKIs的作用靶点表达下降或消失，影响其临床疗效；④研究对象未经生物标志物进行选择。第二种治疗策略是当肿瘤经化疗后得到控制时，应用EGFR-TKIs作为维持治疗。两个三期研究SATURN1和INFORM^[19, 20]^已经表明EGFR-TKIs维持治疗能提高PFS，但是在*EGFR*基因突变患者中使用维持治疗仍存在争议。

若干临床前期和早期研究^[21-23]^评估了化疗间插联合EGFR-TKIs的疗效，结果表明间插联合治疗在*EGFR*突变型和野生型细胞株中均有细胞毒性协同作用，因此间插联合治疗可能是一种有前景的治疗方式。FASTACT-1研究表明化疗间插联合EGFR-TKIs可以提高PFS，而FASTACT-2研究也表明间插联合治疗不仅可以延长PFS和OS，而且还能提高肿瘤缓解率，这些研究证实了间插联合治疗能够增加生存获益。间插联合治疗之所以可以获得更优的临床疗效，原因可能是间插给药方式能避免EGFR-TKIs引起癌细胞阻滞于G_1_周期，化疗药物和EGFR-TKIs间的拮抗作用减弱，从而使化疗的细胞毒性作用最大化。但是FASTACT-2研究还发现，间插联合治疗的临床获益主要体现在*EGFR*突变阳性的患者，对于EGFR野生型或者未知型的患者并不能增加获益。这一结果和本*meta*分析的结果是一致的。

值得注意的是，本*meta*分析纳入的研究使用了不同的EGFR-TKI。对于吉非替尼和厄洛替尼的疗效比较，Burotto等^[24]^和Haaland等^[25]^的研究结果表明两者在晚期NSCLC的一线治疗中，有效率和生存期无明显差异。同时，本*meta*分析的6个研究分别使用了吉西他滨、紫杉醇和培美曲塞等化疗方案，对于三者的优劣对比，我们可以从与黄岩等^[26]^和王强等^[27]^的研究中得到答案。王强等的*meta*分析共纳入6个RCT，包括3, 057例晚期NSCLC患者，结果表明培美曲塞和吉西他滨在有效率和无进展生存期方面无统计学差异，而黄岩等的研究则进一步证实紫杉醇和吉西他滨以及培美曲塞的疗效相似。因此，不同EGFR-TKI和化疗方案对本*meta*分析结果影响较小。

在有效性方面，本*meta*分析表明，和单独化疗相比，间插联合治疗可以提高PFS，同时PFS的亚组分析还表明PFS的临床获益主要发生在女性、年龄 < 65岁、Ⅲb期、腺癌、从不吸烟以及*EGFR*突变的患者。但是，一线间插联合治疗并不能提高患者的OS、ORR和DCR，原因可能和纳入的患者缺乏*EGFR*基因检测，从而未能进行EGFR-TKIs优势人群的选择有关。这一原因在Choi等^[16]^的研究中得到了证实。另外，本*meta*分析虽然证明了化疗间插联合EGFR-TKIs能获得更长的PFS，但具有较高的异质性。为了进一步探索这种异质性，我们根据研究所使用的EGFR-TKIs类型进行了亚组分析。在吉非替尼组，研究间异质性消失，而厄洛替尼组的异质性下降不明显。同时在ORR方面，吉非替尼组和厄洛替尼组的异质性均明显下降，故纳入研究间的异质性和研究所使用的EGFR-TKIs可能有关。这一结果和Burotto等^[24]^以及Haaland等^[25]^的研究结果不一致。

同时，本*meta*分析根据化疗方案进行了亚组分析，结果显示，在ORR方面，吉西他滨组的异质性下降，而在PFS、OS和DCR方面，吉西他滨组的异质性无明显变化。这一结果表明不同化疗方案对*meta*分析结果影响小。此外，本*meta*分析还根据纳入文献的质量分级进行了亚组分析，结果显示，在PFS、OS和ORR方面，“B”级和“C”级组的异质性无明显变化，但是在DCR方面，“B”级组的异质性消失。该结果提示纳入文献的质量分级可能对本*meta*分析结果产生一定影响。因此，为了进一步验证间插联合治疗是否具有更优的生存获益，需要做以下改进：①分别在*EGFR*突变型和EGFR野生型的患者中进行间插联合治疗的大样本研究；②在上述大样本研究的基础上，分别比较不同化疗方案和EGFR-TKIs间插联合治疗的疗效，寻找更优的EGFR-TKI和化疗组合；③严格按照随机分配方法、分配隐藏、盲法、不完整数据报告、选择性发表及其他偏倚来源等六个标准设计试验，降低试验偏倚。

在安全性方面，本*meta*分析表明，化疗间插联合EGFR-TKIs组和单独化疗组的不良反应发生率相似，主要不良反应有皮疹、腹泻、恶心、疲乏和骨髓抑制等。其中间插联合治疗组的皮疹和腹泻的发生率高于单独化疗组，但是多为1级-2级不良事件，经临床处理后可缓解，其余不良发应在两组间无明显差异，因此间插联合治疗并未明显增加不良反应的发生，耐受性良好。

综上所述，化疗间插联合EGFR-TKIs较单独化疗一线用于晚期NSCLC患者是一种可行的治疗选择，尤其是对于非吸烟者和*EGFR*突变阳性患者，值得临床推广。然而，这一*meta*分析存在一些局限性，尚需要在病例选择和治疗方式的设计上加以改进，进行更多大样本、高质量的随机对照试验进一步探索间插联合治疗的潜在优势。
